# m6A RNA Methylation in Systemic Autoimmune Diseases—A New Target for Epigenetic-Based Therapy?

**DOI:** 10.3390/ph14030218

**Published:** 2021-03-05

**Authors:** Anna Wardowska

**Affiliations:** Department of Embryology, Medical University of Gdansk, 80-210 Gdansk, Poland; anna.wardowska@gumed.edu.pl

**Keywords:** epigenetic, autoimmune disease, RNA methylome, m6A, N6-methyladenosine, epidrugs

## Abstract

The general background of autoimmune diseases is a combination of genetic, epigenetic and environmental factors, that lead to defective immune reactions. This erroneous immune cell activation results in an excessive production of autoantibodies and prolonged inflammation. During recent years epigenetic mechanisms have been extensively studied as potential culprits of autoreactivity. Alike DNA and proteins, also RNA molecules are subjected to an extensive repertoire of chemical modifications. N6-methyladenosine is the most prevalent form of internal mRNA modification in eukaryotic cells and attracts increasing attention due to its contribution to human health and disease. Even though m6A is confirmed as an essential player in immune response, little is known about its role in autoimmunity. Only few data have been published up to date in the field of RNA methylome. Moreover, only selected autoimmune diseases have been studied in respect of m6A role in their pathogenesis. In this review, I attempt to present all available research data regarding m6A alterations in autoimmune disorders and appraise its role as a potential target for epigenetic-based therapies.

## 1. Introduction—A Link between Epigenetics and Autoimmunity

Autoimmune diseases (ADs) comprise a wide group of conditions arising from the immune system’s erroneous reactivity. They have become a significant clinical issue through recent years, mainly due to their chronic nature, the prevalence in industrialized populations and high healthcare costs. More than 100 types of autoimmune disorders can be distinguished based on the specificity of the immune reaction and pathogenesis. The general background of all types of the ADs is a combination of genetic, epigenetic, and environmental factors that lead to defective immune reactions and, subsequently, to excessive production of autoantibodies and prolonged inflammation. ADs significantly vary in terms of affected organs and clinical manifestation, as some of the conditions are limited to particular tissues or organs (i.e., multiple sclerosis), whereas others are systemic or disseminated (i.e., systemic lupus erythematosus) [1,2]. Regardless of the affected area, these diseases progress slowly, leading to severe tissue damage at the time of the diagnosis and the onset of appropriate treatment. Moreover, the heterogeneity of symptoms may result in an incorrect initial diagnosis, therefore prolonging the introduction of adequate clinical management. ADs are usually managed by implementing immunosuppressive treatment, including nonsteroid anti-inflammatory drugs, glucocorticosteroids, mycophenolate mofetil, and azathioprine or cyclophosphamide. The general strategy is to target symptoms because the disease’s cure is still out of reach. Due to a long list of unwanted side effects, both scientists and clinicians incessantly search for new therapeutic targets and strategies. Due to the increasing amount of data focused on the epigenetic background of autoimmunity, epigenetic-based treatment has recently become kind of a “hot topic” in terms of autoimmune disorders’ clinical management. There are several epigenetic drugs undergoing clinical trials [3,4], but at present, few of them have been introduced into clinical use (for the management of hematological malignancies) [4,5,6]. Unfortunately, the epidrugs currently available on the pharmaceutical market do not fulfil autoimmunity treatment requirements, mainly due to serious side effects. This review will focus on a “new player” in epigenetic mechanisms, precisely m6A RNA methylation, as both a culprit of autoreactivity and a potential target for epigenetic therapy.

### Role of Epigenetic Alterations in the Pathogenesis of Autoimmune Diseases

During recent years epigenetic mechanisms have been extensively studied, and the putative relationship between altered epigenetic patterns and pathological processes was elucidated [7,8,9]. So far, it is widely known that epigenetics is inevitable in the regulation and maintenance of physiological processes such as cell growth, development, differentiation, and genomic stability [10]. Epigenetics refers to mechanisms that may alter gene expression without changes in the DNA nucleotide sequence. There are three basic modifications, called epigenetic triad, that form the epigenetics foundation: DNA methylation, chromatin remodeling through histone modifications and noncoding RNAs [11]. Dysregulated epigenetic mechanism can introduce gene expression changes causing a disturbance in immune homeostasis and the occurrence of pathological autoimmune processes [8,10,12].

Methylation of DNA is one of the most important epigenetic modifications that can change gene expression by facilitating or hindering transcription factors binding to their target sequences [13,14]. The modification of chromatin availability is based on the addition of methyl group to the deoxycytosine base in CpG dinucleotide to form 5-methyl2′-deoxycytidine (5mC) [14,15,16]. Of high importance is that DNA methylation, catalyzed by methyltransferases (DNMT), is a reversible process [17]. Hence DNA methylation has become an appealing target for future epigenetic-based therapy in autoimmunity and other disorders.

Histone modifications are usually in relationship with DNA methylation, as increased frequency of 5mC in DNA region attracts deacetylation of histones which enhances gene silencing and chromatin compacting. Histones can be subjected to various post-translational modifications, i.e., acetylation, methylation, phosphorylation, ubiquitination, sumoylation, resulting in chromatin remodeling and alterations in the accessibility of DNA to transcription factors [18,19]. Acetylation and methylation of histone residues have been the most effectively studied to date. Both modifications, mediated by different enzymes with opposite functions, are reversible [20,21,22].

Finally, noncoding RNAs (ncRNAs) also exert a considerable impact on the epigenetic landscape of immune cells in systemic autoimmunity. The diversification of ncRNAs is based on their size: microRNA (miRNA) are usually 22–23 nucleotides, whereas long noncoding RNA (lncRNA) are over 200 nucleotides. miRNA can deregulate mechanisms of the innate and adaptive immune response, and this way may interfere with immune homeostasis resulting in the high predominance of autoimmune disorders [13,23,24]. Additionally, lncRNAs are suggested to play critical regulatory roles in a variety of biological processes and diseases [25], even though the knowledge about lncRNA mediated mechanisms is still scarce. It has been stated that lncRNAs may take part in diverse gene-regulatory mechanisms, including gene transcription, RNA splicing, chromatin remodeling, and protein transport [24,26].

## 2. New Epigenetic Player—m6A Modifications of RNA

Alike DNA and proteins, also RNA molecules are subjected to an extensive repertoire of chemical modifications. Over 100 modifications have been identified so far, including 5-methylcytosine (m5C), N6-methyladenosine (m6A), pseudouridine (Ψ), N1-methyladenosine (m1A). Such modifications can be found in mRNA, tRNA, lncRNA, and miRNA [27]. m6A is recognized as the most prevalent form of internal mRNA modification in eukaryotic cells [28].

**Figure 1:**
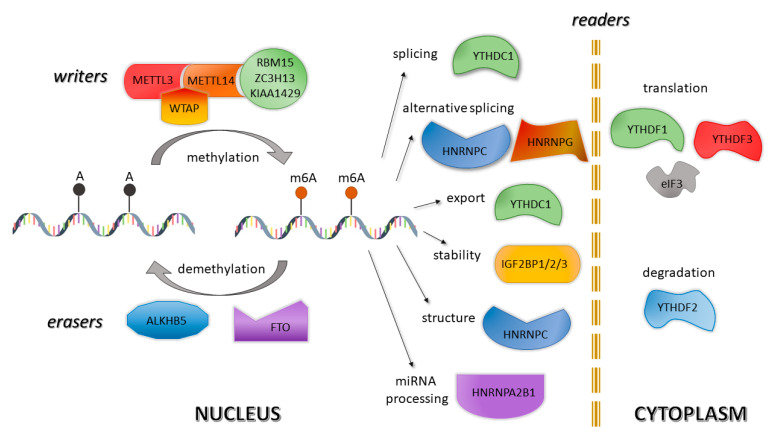
Roles of N6-methyladenosine “writers”, “erasers” and “readers” in regulation of mRNA.

Similar to the modifications of DNA and proteins, also m6A turned out to be a reversible process [29]. This dynamic process of RNA adenine methylation is driven by three primary groups of enzymes responsible for methylation, demethylation and decoding of methylation code, named “writers”, “erasers”, and “readers”, respectively (Figure 1) [30].

The specific RNA methylation process is mediated by a multisubunit methyltransferase enzyme complex composed of three elements: METTL3 (methyltransferase-like protein 3), METTL14 (methyltransferase-like protein 14), and WTAP (Wilm’s tumor-1-associated protein) [31]. This complex recognizes a highly conserved consensus site and specifically methylates only the adenosine’s N6 amino group [32]. METTL3 is a core component responsible for the methyl-group transfer, while its homolog—METTL14—does not exert methyltransferase activity, even though both subunits contain a methyltransferase domain. Therefore, METTL14 works as an RNA-binding platform because it plays a structural and noncatalytic role in substrate recognition, maintaining complex integrity and substrate RNA binding [28]. METTL3 and METTL14 act synergistically to stabilize the complex, which can be subsequently localized into nuclear speckles by the third complex component—WTAP. Moreover, WTAP is believed to modulate the METTL3-METTL14 complex recruitment to mRNA targets because its loss reduces the RNA-binding capability of METTL3 [33,34]. M6A RNA methylation is also supported by several regulatory factors, including RBM15, ZC3H13 or KIAA1429 [35].

There are currently only two demethylases associated with m6A methyl-group removal, and both belong to AlkB family proteins: FTO and ALKBH5. FTO, a fat mass and obesity-associated protein, was the first discovered m6A demethylase that, besides, indicated the reversibility of RNA modifications. The primary function of FTO, localized in the nucleus, is the reduction of m6A levels via oxidative demethylase activity [36,37]. FTO exerts its demethylating activity on m6A in single-stranded RNA [36]. The other demethylase—ALKBH5 (ALKB homolog 5)—also removes the m6A modification of nuclear RNA, but unlike FTO, ALKBH5 can directly catalyze the demethylation of m6A-methylated adenosine [38]. Demethylation by ALKBH5 also can further modulate nuclear RNA export, RNA metabolism and gene expression [39]. FTO and ALKBH5, members of the same protein family (ALKB), share their function but differ in intracellular localization and tissue distributions [40].

The recognition of m6A modifications and subsequent transformation of m6A modifications into diverse functional signals is performed by another class of proteins, termed “readers” [41]. m6A readers/effectors are proteins with the highly conserved YTH (YT521-B homology) domains, including YTHDF1 (YT521-B homology domain family 1), YTHDF2 and YTHDF3 in the cytoplasm and YTHDC1 (YT521-B homology domain containing 1) and YTHDC2 in the nucleus [42]. A recent study revealed that heterogeneous nuclear RNPs (hnRNPs), i.e., HNRNPA2B1, HNRNPC, HNRNPG, can also serve as potential nuclear m6A readers [43].

M6A modifications participate in a variety of functional RNA pathways. They either modulate the structure of the methylated transcript to prevent/induce protein–RNA interaction or induce subsequent reactions through direct recognition by m6A binding protein [44]. The “readers” from the YTH family preferentially bind to m6A, with YTHDF1 primarily affecting the gene translations by m6A modification, YTHDF2 affecting their degradation and YTHDF3 exerting impact on splicing [45]. The nuclear protein HNRNPC directly binds nuclear m6A-methylated transcripts, thus regulating their alternative splicing [46,47]. Additionally, translation factor eIF3 (eukaryotic initiation factor 3), a large multiprotein complex, has been identified as a novel m6A reader [48]. eIF3-mediated translation is initiated by binding to the m6A site in the 5′-UTR of mRNA. Meanwhile, the family of IGF2BPs (insulin-like growth factor 2 mRNA-binding proteins) stabilize the target gene and the corresponding translation [49].

### 2.1. Methods for m6A Detection

Even though RNA methylations were initially discovered in the XX century (i.e., m6A in 1975 [50], m5C in 1958 [51] or m1A in 1961 [52]), the exploration of their function in health and disease was significantly limited due to the lack of available methods for its detection. Owing to high-throughput sequencing development, the physiological importance of RNA methylation could be studied [53]. As m6A modifications do not change the base pair nature, the direct diversification from regular bases is problematic. The m6A detection methods can be distinguished, based on their precision, into three main groups: semiquantitative, quantitative and procedures allowing the detection of precise locations [53]. Semiquantitative methods include dot blot (or slot blot) technology [54,55], methyl sensitivity of MazF RNA endonucleases [56] and immune-Northern blot [57,58]. Each of them allows the confirmation of m6A modification presence in the analyzed RNA but is far from quantitation or precise determination of m6A localization. The RNA photo-crosslinkers and quantitative proteomics rely on the tendency for UV-induced photochemistry of nucleobases [59]. This method utilizes specific RNA probes containing three elements: m6A molecules, a photo-crosslinker and streptavidin for protein enrichment. The second of the quantitative techniques called the electrochemical immunosensor method, uses the specific anti-m6A antibody, whose detection relies on silver nanoparticles and SiO2 nano-spheres with amine-polyethylene glycol 3-biotin. The vector machine method was developed for quantitative analysis of m6A sites in *Arabidopsis thaliana*. The combination of anti-m6A antibodies and high-throughput sequencing allowed sufficient identification of specific m6A sites [60,61]. The major limitation of both semiquantitative and quantitative methods is the inability to precisely and directly identify the m6A modification sites.

With the development of next-generation sequencing technologies, it became possible to map m6A residues within RNA fragments. Me-RIP-Seq (methylated RNA immunoprecipitation sequencing) is a novel technique for identifying m6A sites in mammalian RNA. This combination of ChIP-Seq and RNA-Seq emerged in 2012, starting a new era of m6A research [62]. The mRNA samples are at first randomly fragmented into 100–150 nucleotide segments, which are subsequently incubated with an anti-m6A polyclonal antibody. Following immunoprecipitation, the enriched m6A-containing pooled RNA and input RNA control are subjected to deep sequencing [62]. Regardless of easiness and efficiency, the Me-RIP-Seq resolution of approximately 200 nucleotides is rather impractical to identify m6A positions precisely [63]. SCARLET (site-specific cleavage and radioactive-labeling followed by ligation-assisted extraction and thin-layer chromatography) provides the precise location of m6A at any site in mRNA/lncRNA with single-nucleotide resolution [64]. This precision is obtained due to the usage of RNase H. This enzyme’s addition to the total RNA sample is followed by radiolabeling using ^32^P and subsequent splint ligation to DNA oligonucleotides by DNA ligase. Only ^32^P-labeled sites avoid RNases T1/A digestion and can be further analyzed with thin-layer chromatography (TLC) [64]. The significant disadvantages of the SCARLET technique are the incapability of high throughput screening and laboriousness [63]. Another recently developed method—m6A-LAIC-seq (m6A-level and isoform-characterization sequencing)—can quantify m6A presence on a transcriptome-wide level [65]. The excess anti-m6A antibody is used in a full-length RNA immunoprecipitation. Then the sample is treated with External RNA Controls Consortium (ERCC), and the internal standards are supernatant (m6A-negative fraction) and eluate (m6A-positive fraction). The final high throughput sequencing allows for m6A site detection and compares alternatively spliced isoforms between m6A-positive and m6A-negative fractions [65].

To overcome some common m6A methods limitation, a modified cross-linking and immunoprecipitation (CLIP) based approach has been employed. This technique relies on the induction of specific mutations during reverse transcription via UV–cross-linking of the anti-m6A antibody to methylated RNA [66]. M6A CLIP and miCLIP (m6A individual-nucleotide-resolution cross-linking and immunoprecipitation) can accurately locate m6A loci in the whole transcriptome at a single-nucleotide resolution level without any pretreatment of cells [66]. Several variants for the CLIP approach have been recently established, including enhanced CLIP (eCLIP) or m6A-eCLIP (meCLIP). Compared to the basic CLIP method, the new protocols are technically simplified and yield higher numbers of identified sites [67]. The selected m6A detection methods are presented in Table 1 and reviewed in [53,68,69].

Identifying specific m6A residues within RNA transcripts is of great value in further comprehending m6A biological function and utility in various disease management. The development of the above-listed methods was a groundbreaking discovery in RNA methylome research. Several of these techniques, including Me-RIP-Seq, led to the identification of the downstream genes and mutation sites [70,71]. Therefore, we must unceasingly elaborate on technologies with higher precision of m6A detection and reduced laborious steps.

### 2.2. m6A in Health

Based on its prevalence, evolutionary conversation and nonrandom distribution, the role of RNA modification suggests its importance as an epitranscriptomic mark that can modulate almost all aspects of RNA transcript metabolism, i.e., splicing, stability, structure, translation, and transport [77]. Biological consequences of these RNA modifications are visible on three levels of organization: molecular, pathway or cellular and physiological. The impact on the molecular level focuses on the changes on the target RNA’s structural level and subsequent transmission to RNAs function and fate. These changes include alternations of mRNA splicing pattern, nuclear export, translation and stability. Each of these processes may affect the final realization of the mRNA message, thus influencing cell metabolism and, finally, the organism’s physiology. As to the consequences of RNA methylations at the pathway or cellular level, recent findings suggest that these modifications may significantly influence the maintenance and differentiation of mouse embryonic stem cells [78]. The role of m6A RNA modification in mESCs pluripotency regulation proved to be complicated and depended on the state of these cells [79].

Moreover, the cell type-specific RNA methylome appeared to be regulated by microRNA. The latest studies emphasize that RNA methylation modifications exert control over the cellular development fate, thus promoting appropriate and complex responses to developmental signals. By controlling gene expression and cellular pathways, m6A is involved in distinct biological processes, including immune responses. It has been proven that brain tissue is highly enriched in m6A, and this modification plays a vital role in the development of the nervous system [80,81,82]. The proper development of the brain tissue can be severely affected by the altered expression of m6A writers or readers [83]. Animal studies revealed that one of the m6A readers—YTHDF1—is involved in the process of learning and memory. YTHDF1 specifically recognizes m6A, thus facilitating the translation of targeted transcript in the hippocampus in response to neuronal stimuli [84]. Additionally, FTO has also been strongly associated with a variety of brain functions [85], including learning and memory abilities as well as behavioral training [28,86]. M6A also seems important in the process of gametogenesis, as spermatogenic cells at different developmental stages are abundant in this RNA modification [87]. The available data imply that m6A-dependent RNA translation may control the late stage of spermatogenesis [87], as well as regulate spermatogonial stem cell differentiation and meiosis initiation [38,88,89,90].

Undoubtedly, m6A RNA modification is one of the key players in immunity, as its dysregulation results in a wide spectrum of pathological states. It has been demonstrated that m6A plays a vital role in the T-cell homeostasis through activation of IL7/STAT5/SOCS signaling in naïve T cells, thus initiating their reprogramming for cell proliferation and differentiation [91,92]. M6A modification is also involved in innate immunity. A recent study revealed that Mettl3-mediated mRNA m6A methylation promotes dendritic cell (DC) activation and function [93]. These results confirm an assumption that RNA methylome can regulate immune response on several levels, thus contribute to both health and disease.

### 2.3. m6A in Disease—Alterations in Autoimmune and Related Disorders

The increasing number of studies anticipate the significance of m6A modification in human diseases [94,95,96,97]. It is believed that reversible RNA methylation underlies diverse consequences at the physiological level and is associated with a variety of human diseases. Currently, the primary research field of m6A modification is oncology [98,99]. The changes in RNA methylome are believed to mainly promote or inhibit tumor production by regulating the mRNA levels of related oncogenes or suppressor genes. Of significance is that the same components in different types of tumors do not play the same role; sometimes they even oppose each other. Research data regarding the role of m6A in cancer onset and development have been widely reviewed in many other scientific papers [100,101,102,103,104].

As m6A modifications are engaged in neurodevelopment, they may greatly impact the proper functioning of the nervous system. Indeed, some associations between malfunction of m6A pathway signaling, nerve injury and malformation were confirmed [105,106]. Studies on axonal regeneration of adult mouse dorsal root ganglion revealed a crucial role of m6A methylation in normal physiology and responses to pathological stimuli in the adult mammalian nervous system [105]. Genetic variants of enzymes engaged in m6A methylation may also affect mental health. For instance, different genetic variants of FTO, aside obesity promotion [107], can decrease the risk of depression [108,109] and be involved in modulating the risk of ADHD (attention-deficit/hyperactivity disorder) [110]. Interestingly, there is a possible link between transcripts modified by m6A and mental disorders, including autism and schizophrenia [111]. Several other psychiatric disorders, such as Alzheimer’s disease or Parkinson’s disease, were associated with altered m6A modifications [112,113,114]. Moreover, many research papers report that m6A methylome exerts its role in nutritional physiology and metabolism [115,116], osteogenic differentiation [117,118] or cardiovascular system homeostasis [119,120].

The m6A methylation is presumed to play an important role in the functioning of the immune response, both in healthy individuals and patients suffering from various disorders [77]. Changes in mRNA methylome can strongly influence T cells function and development in the thymus, leading to aberrant immune responses. The decrease of T cell proliferation and differentiation has been linked to the absence of METTL3 [91,92]. The depletion of METTL3 in Tregs results in increased stability of the SOCS gene, therefore blocking the transduction of cytokine signaling in the IL-STAT5 pathway [92]. The blockade of METTL3 is likely to be a crucial factor in regulating immune homeostasis and the decreased induction of various autoimmune diseases [121]. In dendritic cells, the METTL3 depletion impairs phenotypic and functional maturation of DCs, manifesting through reduced expression of the costimulatory molecules (CD40, CD80) and limited secretion of IL2. These METLL3-associated alterations in DCs activity affect their ability to stimulate T-cell responses in vitro and in vivo [93]. Alike, YTHDF1 has been reported to influence certain immune transcripts translation in DCs, hence modifying DCs interaction with T cells [122]. With the increasing knowledge about RNA methylome and its impact on living organisms’ physiological processes, the interests in unveiling its role in human diseases are incessantly growing. Autoimmune diseases, next to cancer, have moved to the forefront in the field of epitranscriptomic research. The available data about m6A modification may suggest some association between RNA methylation levels and autoimmunity initiation and progression [123].

#### 2.3.1. Systemic Lupus Erythematosus

Systemic lupus erythematosus (SLE) is one of the most investigated but still mysterious autoimmune disorders. Due to its pathomechanism, SLE is considered an archetypic autoimmune disease, characterized by intermittent episodes of symptoms augmentation imposing immunosuppressive treatment [11,124]. The pathogenic process is associated with antinuclear antibodies (ANA) formation, decomposition of immune complex (IC) in the affected organism, sustained immunological response, resulting in multiple tissue damage. Almost any part of the human body can be affected by an ongoing autoimmune process; therefore, the clinical manifestation of SLE is of huge diversity [124,125]. The complexity of SLE background, triggers, pathomechanisms, and outcomes is highlighted by several sets of classification criteria that had to be developed (i.e., ACR, SLICC, SLEDAI-2K, EULAR, and others) [126]. Classification criteria, despite their indisputable importance in difficult diagnosis and treatment, reflect our current understanding of the disease [127].

The etiology of SLE is highly complex due to overlapping factors leading to the tolerance breakdown. New perspectives, enabling the solution of this puzzling issue, appeared along with the emergence of epigenetic studies, suggesting the existence of interplay between the cellular, environment, epigenetic factors, and genome [10]. The correlation between epigenetic changes and the pathogenesis of SLE has already been proven [13,128,129]. Lupus was one of the first autoimmune diseases studied to demonstrate the influence of epigenetic alterations on the disease course [130,131,132,133].

With the increasing knowledge about RNA methylome and its impact on physiological processes in living organisms, the interests in unveiling its role in human diseases are incessantly growing. The available data about m6A mechanisms may suggest some association between RNA methylation levels and SLE initiation and progression. The first potential link between RNA methylome and the initiation and progression of SLE is the aberrant expression of proteins engaged in RNA methylation occurrence [134,135]. Such alterations would affect the global RNA methylation levels leading to changed expression of crucial immune-related genes and autoimmunity development. It has already been proven that transcripts associated with the production of type I interferons and the differentiation of T cells can be altered through changes in m6A RNA methylation [136]. This could somehow explain abnormal interferon levels and Th17 frequency observed in lupus patients [137].

Unfortunately, little evidence has been published so far [134,135] (Table 2). The first available data were focused on the mRNA expression of m6A writers (METTL3, METTL14, and WTAP), erasers (FTO and ALKBH5) and readers (YTHDF2) in peripheral blood mononuclear cells (PBMCs) from lupus affected patients [134]. The qPCR analyses revealed altered expression of the examined genes, with the general decreasing tendency in patients compared to healthy controls. Moreover, interesting relations were detected between gene expression and a set of clinical features. The decreased expression of ALKBH5 was associated with CRP (positive association), neutrophil and lymphocyte percentage and ratio (negative association), C3 levels (positive association), and fever. The expression of METTL14 in PBMC from SLE patients displayed a negative correlation with white blood cell count (WBC) and monocyte count. Additionally, mRNA expression of YTHDF2 turned out to be associated either positively (lymphocyte percentage and C3 levels) or negatively (neutrophil–lymphocyte ratio) with several clinical SLE features. The authors suggested the link between the decreased levels of METTL14, ALKBH5 and YTHDF2 expression in PBMCs and SLE pathogenesis. With further statistical analysis, they confirmed that the reduced amount of mRNA for YTHDF2 could be regarded as a risk factor for SLE and utilized as a routine clinical parameter [134].

Another report [135] confirmed the involvement of ALKBH5 in the pathogenesis of SLE, as its levels were significantly decreased compared to patients with other diseases (rheumatoid arthritis, hepatitis B virus-infected patients, and tuberculosis patients [135]). The level of ALKBH5 mRNA in SLE patients’ peripheral blood was also negatively associated with the production of autoantibodies (anti-dsDNA). Moreover, the expression of ALKHB5 proved to be related to some clinical symptoms like rash, ulceration and leukopenia. The authors also acknowledge the role of other enzymes engaged in the m6A methylation pathway, METTL3, WTAP and FTO as potential autoreactivity collaborators in SLE [135]. All the data mentioned above reveal a considerable shortage of general knowledge about m6A and an urging need for further studies on RNA methylome alterations in SLE pathogenesis.

#### 2.3.2. Rheumatoid Arthritis

Rheumatoid arthritis (RA) is an autoimmune musculoskeletal disorder primarily affecting connective tissue, precisely synovial joints, but finally leading to systemic manifestation. The ongoing chronic autoinflammatory process gradually leads to damage of articular surfaces and extraarticular manifestation, finally attacking the skin, lung, heart, and eyes [138,139,140,141]. In terms of immunological background, the process of synovial inflammation and extraarticular autoaggression is triggered and sustained by aberrant reactivity of both innate and adaptive immunity. The dysfunction of synovial tissue allows invasion of activated macrophages, granulocytes and lymphocytes, perpetuating the destruction process. Even though RA presents a large number of autoantibodies (for example, rheumatoid factor—RF and anticitrullinated protein antibody—ACPA) [142], helpful in diagnosis, T cells exaggerate the autoimmune process through a broad spectrum of produced proinflammatory cytokines [143,144].

The etiology of RA remains unknown but is considered a combination of genetic predisposition and epigenetic factors. Genetic heterogeneity does not explain all the mechanisms behind RNA onset and progression. Moreover, it has been suggested that the pathogenesis of early and late RA stages differ; therefore, do not involve the same pathomechanisms [145]. The immunological mechanisms seem to be decisive in the early period of the disease. In contrast, the advanced/refractory period of RA appears to be more associated with the genetic and epigenetic factors [141]. The latter statement generates a new field of epigenetic research and a novel epigenetic-based therapy quest. It was confirmed that autoimmune-mediated inflammation in RA is simultaneously under genetic [146] and epigenetic regulation [147]. Epigenetic alterations in DNA have been studied in both peripheral blood cells [148] and rheumatoid arthritis synovial fibroblasts (RASFs) [149]. These alterations affect the expression of immune-related genes, thus influencing immune response [150,151,152]. Epigenetic factors associated with DNA in RA have already been revised in detail elsewhere (further recommended reading: [141,144,153,154]).

The relationship between RNA-associated epigenetic changes have been recently proposed to be another layer of epigenetic regulation that might unravel the mystery of RA pathogenesis [155]. As it is a new pathway of epigenetic studies, still in its infancy, the available data are relatively rare. Nevertheless, these reports seem informative and encouraging for scientists uncovering the link between RNA methylome and RA (Table 2). The genome-wide association study performed by Mo et al. [156] identified m6A-associated SNPs (m6A-SNPs) that affected the progression of the disease. The authors detected a set of unique m6A-SNPs in Asian (9 SNPs) and European (32 SNPs) patients with RA. In total, 27 m6A-SNPs were verified to effect expression of 24 local genes in immune cells, including monocytes, CD8 T cells, B cells, regulatory T cells (Tregs), and activated natural killer (NK) cells and synovial tissue. Most of the detected SNPs were in the MHC region, but fine-mapping of association signals in this region is currently out of reach. Additionally, SNPs in the m6A regulators encoded genes (METTL3, METTL14, WTAP, FTO, and ALKBH5) were also examined. Five SNPs in METTL3, 1 SNPs inMETTL14, 1 SNPs in WTAP, 160 SNPs in FTO, and 21 SNPs in ALKBH5 were found for Asian or European populations. These results indicate the potential role of m6A-SNPs in the course of RA [156].

The other study undertook the elucidation of the function and potential mechanism of METTL3 in RA pathogenesis [157]. The expression of METTL3 was evaluated both in peripheral blood from RA patients and in LPS-stimulated macrophage cell line (THP-1). RA patients were characterized with increased transcription of METTL3, which positively corresponded with CRP (C-reactive protein) and ESR (erythrocyte sedimentation rate), two common markers for RA activity. Interestingly, LPS stimulation of macrophages led to enhanced expression and biological activity of METTL3. On the other hand, overexpression of METTL3 attenuated the inflammatory response firstly induced by LPS. Based on these results, it is stated that the effect of METTL3 on LPS-induced inflammation in macrophages was dependent on NF-κB. In conclusion, the authors claim METTL3 is a potential biomarker for the diagnosis of RA [157].

The last of the published reports are focused on the expression of the key enzymes of m6A methylation modification: ALKBH5, FTO, and YTHDF2 [158]. The transcript levels of these three m6A-associated enzymes proved to be significantly decreased in patients suffering from RA. However, the expression of ALKBH5 was affected by regular treatment and increased in patients subjected to appropriate drug therapy. The other two enzymes’ expressions proved to be correlated with some common markers for RA disease activity. The mRNA expression of ALKBH5 was significantly increased in RA patients that received regular treatment. The mRNA expression of FTO was associated with DAS28 (disease activity score 28), C3 levels, IgG, and LMR (lymphocyte-to-monocyte ratio) (LMR). At the same time, the transcription level of YTHDF2 revealed a relationship with RBC (red blood cell count), lymphocyte percentage, neutrophil percentage, NLR (neutrophil-to-lymphocyte ratio), and LMR. Moreover, peripheral blood global m6A content was significantly increased in patients with RA compared to healthy individuals. In conclusion, the authors acknowledge the critical role of ALKBH5, FTO, and YTHDF2 in the RA’s pathogenesis and disclose these compounds as promising biomarkers for RA [158].

#### 2.3.3. Psoriasis

Psoriasis (Ps) is a chronic, immune-mediated skin disorder accompanied by various extracutaneous comorbidities, including psoriatic arthritis, uveitis, metabolic disorders, and cardiovascular diseases [159]. With its broad spectrum of subtypes and manifestations, this condition is considered a noncommunicable, painful, disfiguring, and disabling disease for which there is no effective cure [160]. The most common form of Ps—psoriasis vulgaris—manifests through red, well-demarcated plaques and silvery dry scale located predominantly on elbows, knees, scalp, navel, and lumbar area [161]. Moreover, Ps may involve oral mucosa, soles of the feet, palms of the hands, and nails [162]. The enhanced systemic inflammatory responses induced by Ps can also cause metabolic abnormalities, cardiovascular diseases [163,164] and irreversible joint destruction. One of the Ps hallmarks is the hyperproliferation of premature keratinocytes and an incomplete cornification that results in a thickened epidermis [165]. Another characteristic feature is the immune cell infiltration, including T cells, neutrophils, dendritic cells, and macrophages [Lowes MA 2014]. Th17 and Th1 cells seem crucial for Ps’ pathogenesis, as they are the major source of cytokines (IL17, IL23), exaggerating inflammation in the skin. The IL23/IL17 axis, along with TNF, TGFβ, IL6, IL8, IL21, and VEGF secreted by other immune cell populations and activated keratinocytes, contribute to Ps’ formation of epidermis phenotype and inflammatory loop in the psoriatic lesion [159,165].

Concerning Ps etiopathology, genetic susceptibility and exposure to certain environmental factors elicit disease phenotype. The genetic predisposition is associated with several HLA-related genes, including HLA-B*39, HLA-B*07, HLA-B*27, and HLA-B*38 alleles already described as specific risk factors for Ps [166,167]. Genetic studies in psoriasis-affected families allowed the identification of several psoriasis susceptibility loci (PSORS) located inter alia in the MHC region [168]. PSORS1 is the first discovered susceptibility loci of Ps and considered the strongest heritable risk factor of this disorder [169]. HLA-Cw6, identified within PSORS1 loci [170], encodes the MHC class I molecule responsible for antigen presentation to T cytotoxic cells [171]. Therefore, it is hypothesized to alter autoantigen presentation resulting in erroneous activation of the immune system in Ps [172]. Not only antigen presentation is affected in Ps, but the inflammatory pathway mediated by NF-κB is significantly altered in primary human keratinocytes. The culprit is associated with specific mutations in CARD14 genes, a scaffolding protein involved in NF-κB activation [173,174]. Not only genetic factors but also environmental triggers are responsible for Ps pathogenesis. The extensive overview of Ps development factors can be found in the following reviews by Zheng et al., Perera et al. and Takahashi and Iizuka [159,162,165].

Epigenetics research in psoriasis has become an expanding field in recent years and proved to impact our disease understanding [175,176,177]. Several genome-wide association studies revealed epigenetic alteration in blood cells [178,179] and affected skin [180,181,182,183] in psoriasis-affected individuals. Although the epigenetic triad, DNA methylation, histone modifications and noncoding RNAs have been extensively studied in the Ps, RNA methylome has not been fully elucidated yet. Hitherto only one research paper has been published on this topic [160] (Table 2). The authors performed m6A transcriptome-wide profiling in three kinds of skin tissue: involved psoriatic skin (PP), uninvolved psoriatic skin (PN), and healthy control skin samples (NN). M6A is highly conserved across psoriasis vulgaris and healthy controls, but several differences among PP, PN, and NN appeared. The comparative analysis of acquired MeRIP-Seq data revealed that psoriatic skin samples and healthy samples contained the fewer shared m6A peaks. In contrast, uninvolved psoriatic skin and control group carried the most. Moreover, the differentially methylated RNAs (DMRs) analysis showed greater disparities between psoriasis affected skin (PP) and control samples than between psoriasis unaffected skin (PN) and controls. PP skin samples were characterized with a higher number of hypomethylated DMRs than hypermethylated. The latter accumulated in DCs and 3UTRs and proved to be particularly associated with response-associated terms, cytokine production, and olfactory transduction. Contrary, the hypomethylated transcripts in PP were mainly linked with development-related processes and the Wnt signaling pathway. The authors confirmed that altered m6A methylation pattern affected gene expression. IL17A and TNFα, the key genes of the TNFα/IL23/Th17 axis in psoriasis, were significantly upregulated in psoriasis skin samples compared to healthy samples. Therefore it can be stated that the upregulation of gene expression was often accompanied by the upregulation of m6A methylation regardless of the peak position, suggesting a possible positive relationship between the extent of m6A methylation and the mRNA levels in psoriasis and other autoimmune diseases [160].

#### 2.3.4. Multiple Sclerosis

Another disease of autoimmune origin is a neurological disorder—multiple sclerosis (MS)—characterized by immune-mediated myelin destruction. MS symptoms are restricted to a particular body region as an organ-specific autoimmune disease—the central nervous system [184]. Neurodegeneration observed in this disorder, is a result of demyelination and loss of oligodendrocytes [185,186]. Moreover, such lesions are irreversible and manifest in patients as incoordination, sensory loss, weakness, changes in bladder capacity and bowel function, fatigue, and cognitive impairment [187]. Thus, MS has a severe impact on patients’ comfort of life, their ability to work and participate in their communities’ social life, finally leading to socioeconomic dependence on their families and/or a public institution such as social welfare [188,189]. The disease’s etiology has not been elucidated yet and requires further evaluation of the interaction between genetic predisposition and environmental factors.

Concerning immunology background, MS is believed to be caused by the inappropriate activation of autoreactive CD4+ T cells [184]. Therefore, this subpopulation of helper T cells is considered a major culprit and propulsion of the autoimmune process. Nevertheless, DCs also appear to be responsible for triggering autoaggression via the presentation of amino acids similar to myelin peptides synthesized in the CNS. Upon this activation, CD4+ T cells start to secrete IFNγ, which recruits other immune cells in the periphery, including cytotoxic T cells, B cells, along with monocytes [190,191]. These proinflammatory cells infiltrate the blood–brain barrier and consequently exacerbate the inflammatory process triggered by the reactivated CD4+ T cells [192,193]. T cells, both CD4+ and CD8+, are responsible for astrogliosis [194] and microgliosis [195], as well as oligodendrocytes destruction and neuronal death [196], respectively. B cells and monocytes contribute to local inflammation by reactivating CD4+ T cells [197], while B cells on their own aggravate myelin sheath damage by producing antibodies against CNS self-antigens [198].

The genetic factor in the pathogenesis of MS has been widely confirmed [199]. HLA-DR*B1, located in the short arm of chromosome 6 [200], is the strongest genetic risk factor [Simon K 2010]. However, only 27% of MS heritability can be associated with the genetic variant of the HLA system [201]. Therefore, the environmental, thus epigenetic, factors gain more interest as prominent contributors to the pathogenesis of MS. The extensive summary of differentially methylated regions in the course of MS can be found in the review by Celarian and Tomas-Roig [195].

Heretofore, only one scientific paper regarding m6A RNA modification in MS has been published [202] (Table 2). Mo et al. performed an integrative analysis of DNA methylation and gene expressions data that allowed the identification of potential genetic and epigenetic factors behind MS pathogenesis. In this research, N6-methyladenosine was studied as an element of SNPs with specific functions since m6A-SNPs may influence m6A by changing the RNA sequences of the target sites or key flanking nucleotides [203]. The authors identified 13 m6A-SNPs, including rs923829 in METTL21B and rs2288481 in DKKL1 gene, that were significantly associated with MS. It is hypothesized that these two m6A-SNPs act through the regulation of METTL21B and DKKL1 gene expression, as confirmed in the HaploReg database. Moreover, the authors validated these associations in peripheral blood samples of a small cohort of Chinese individuals, proving the m6A-SNPs potential role as culprits of MS [202]. Further research is needed to fully elucidate the impact of the m6A RNA machinery on MS onset and propagation.

Unfortunately, the above-presented reports are the only available data regarding m6A role in autoimmune disease pathogenesis, at least at the time of this literature review. It is an obvious conclusion that m6A is an important factor in shaping immune response both in health and disease and seems to be one of the crucial culprits of autoreactivity. Therefore, following the path of RNA methylome research may bring us closer to unravelling the mystery of autoimmunity along with the identification of new therapeutic targets.

## 3. Epigenetic-Based Therapy in ADs—m6A as a New Target

In recent decades, epigenetics emerged as the link between the genetic and environmental factors underlying the phenotype’s expression in health and disease. Therefore, a surge for potential epigenetic targets in the therapy of various disorders is a logical consequence of epigenetic mechanisms research. With the lack of effective cure in most cases, autoimmune disease is disclosed as the first line of epidrugs targets. Nevertheless, not much has yet been done in this area, even though several classes of epigenetic drugs have already been designed, validated or even approved in humans (reviewed in [3,4,204]). It is believed that epigenetic therapy for ADs can be based on drugs that alter aberrant DNA methylation, histone modification or miRNAs expression in patients with autoimmune disorders. Table 3 presents selected epidrugs with potential application in autoimmunity. Azacytidine (5-azaC), the first developed DNA methyltransferase inhibitor, has proved to be beneficial in several models of autoimmune diseases, including mouse models MS [205], RA [206], and in the clinical treatment of SLE [207]. 5-Aza-dC, or decitabine, chemically similar to 5-azaC, was also successfully evaluated in murine models of induced rheumatoid arthritis [208] and multiple sclerosis [209]. HDAC inhibitors, including FDA approved Trichostatin A or Vorinostat, have also proved effective in autoimmune disease models, both human and mice, in terms of their potential clinical application [210,211,212,213,214,215] (Table 3).

The therapeutic potential of m6A modification can be plausibly predicted, mainly based on currently available data from oncology research. Due to the findings confirming the role of m6A RNA methylation in the control of cancer onset and progression, it is suggested that targeting m6A modification could provide a potential therapeutic target for different human cancers. Particularly, writers and erasers seem to be the most promising therapeutic targets since small molecules can modulate their activity.

Currently, many studies are focused on the development of FTO inhibitors because through demethylation of m6A RNA sites; such inhibitors may influence multiple mRNA related processes, including transcript stability, alternative splicing, mRNA translocation, and protein translation [216,217,218]. The oncometabolite R-2 hydroxyglutarate (R-2HG), able to target FTO/m6A/MYC/CEBPA signaling, restrained leukemia cell proliferation, cell cycle and induced cell apoptosis [217]. Two other compounds, meclofenamic acid (MA) and *N*-(5-Chloro-2,4-dihydroxyphenyl)-1-phenylcyclobutanecarboxamide, have been recently identified as selective inhibitors for FTO by competing with FTO binding for the m6A containing nucleic acid [219,220]. The recently developed FTO inhibitors, FB23 and FB23-2, proved to selectively suppress proliferation of and promoted AML cells apoptosis in vitro, leading to significant inhibition of human AML progression in xenotransplanted mice [218]. Direct binding of FTO protein and blocking its catalytic pocket was detected as a mechanism of action of compounds referred to as CS1 and CS2. Such interaction of these inhibitors with FTO resulted in a strong antitumor effect in multiple types of cancers [221]. Additionally, MO-I-500, another selective FTO inhibitor, proved its effectiveness in repressing the proliferation of triple negative breast cancer cells [222]. Although these inhibitors are promising, some limitations have to be taken into account. In some cases, the selectivity is not high enough to prevent the suppression of other important enzymes [223,224].

The m6A demethylases inhibitors are the most widely studied compounds since with the other m6A modulators the situation is much more complicated. Contradictory results obtained from studies on various cancer types suggest that m6A modification may exert a distinguished impact on cellular mechanisms depending on the cell state. For instance, m6A reader METTL3 shows diametrically opposite regulatory effects in different cancers and may exhibit alternative activities independent of its catalytic properties [31]. Interestingly, immunological studies revealed that METLL3 could modulate several immune cell populations’ activities, including T cells and DCs [91,93]. The activity of this m6A writer upregulated the expression of costimulatory molecules (CD40, CD80) and IL-12, thus promoting DCs’ activation and function [93]. Moreover, neoantigen-specific immunity was shown to be regulated by YTHDF1 in an m6A-dependent manner [122]. Mice classical DCs, deficient in YTHDF1, enhanced the cross-presentation of tumor antigens and the cross-priming of CD8+ T cells in vivo. In this study, transcripts encoding lysosomal proteases were marked by m6A and subsequently recognized by YTHDF1. This interaction resulted in the inhibition of cathepsins, which in turn markedly enhanced cross-presentation of the wild-type DCs. Another interesting observation regarding immune checkpoints was described in this paper. The therapeutic efficacy of PD-L1 checkpoint blockade was enhanced in YTHDF1-deficient mice DCs [122]. Additionally, the depletion of FTO proved beneficial in cancer models, as it led to increased RNA decay through the m6A reader YTHDF2 [225]. All these data suggest that targeting m6A regulators may promote anticancer therapies. However, it also provides an insight into the mechanism used by m6A modification in the regulation of the immune system. It has already been proven that m6A methylation can control T cell homeostasis by targeting the IL-7/STAT5/SOCS signaling pathways [91]. M6A seems to be a suitable and promising factor fitting in this exciting research field with promising clinical implications. It is now time to look closer at the achievements in oncology and try to translate them to the immune dysregulation in autoimmunity. Considering the above-presented data regarding the alteration of m6A RNA methylome in autoimmune disease affected patients, we can state that the m6A role in the pathology of Ads will be clarified in the near future. Therefore, we can also expect to utilize compounds reprogramming the epigenetic m6A landscape to successfully combat chronic autoimmune diseases. However, before we go that far, further studies on the m6A mechanism are inevitable for successfully identifying m6A regulators in autoimmune disorders.

## 4. Conclusions

N6-methyladenosine (m6A) is considered one of the most ubiquitous and abundant mRNA methylation modifications in eukaryotes. Since its discovery in the mid-70s of the 20th century, it focuses on scientific interest as a potential explanation and background of both physiological and pathological processes in the human body. This reversible RNA modification has been widely associated with the proper development and differentiation of organisms and biological functions. However, its dysregulation results in severe pathology, including cancer progression, metabolic diseases or incorrect immune system activation. Even though autoimmune disorders comprise one of the most important health issues worldwide, not much has been done to evaluate the role of m6A in ADs pathogenesis. In this review, I presented all available data on the topic, trying to attract scientists’ attention to the role of RNA methylome in autoimmunity. All the m6A reports reviewed in this paper unquestionably stress the importance of m6A pattern in the onset and propagation of systemic and organ-specific diseases like systemic lupus erythematosus, rheumatoid arthritis, psoriasis, or multiple sclerosis. Still, much has to be done to prove and confirm m6A as a potential biomarker and therapeutic target in the currently neglected autoimmune disorders.

## Figures and Tables

**Table 1:** Selected methods of m6A detection.

Method Name	Abbreviation	Method Principle	Sensitivity	Ref.
Dot blot technology	-	Semiquantitative antibody-dependent identification on a membrane	Medium	[54,55]
Immune-Northern blot	-	UV-crosslinking followed by detection with specific antibody on the nylon membrane	Medium	[57,58]
Methyl sensitivity of MazF RNA endonuclease	MAZTER-Seq	Restriction with endonuclease that cuts methylated but not unmethylated nucleotides	High	[72]
m6A-sensitive RNA-endoribonuclease-facilitated sequencing	m6A–REF-Seq	Restriction with endonuclease that cuts methylated but not unmethylated nucleotides	High	[73]
Site-specific cleavage and radioactive-labeling followed by ligation-assisted extraction and thin-layer chromatography	SCARLET	Restriction with endonuclease that cuts methylated but not unmethylated nucleotides	High	[64]
N6-methyladenosine sequencing	m6A-seq	Immunoprecipitation with m6A-specific antibody	Medium	[74]
Methylated RNA immunoprecipitation sequencing	MeRIP-Seq	Immunoprecipitation with m6A-specific antibody	Medium	[62]
m6A-level and isoform-characterization sequencing	m6A-LAIC-seq	Immunoprecipitation with m6A-specific antibody	Medium	[65]
Cross-linking immunoprecipitation	miCLIP, eCLIP, m6A-CLIP	Crosslinking and immunoprecipitation with m6A-specific antibody	High	[66,67,75]
FTO-assisted chemical labeling method	m6A-SEAL-Seq	FTO-assisted oxidation to hm6A for biotin labelling to immunoprecipitation	High	[76]

**Table 2:** Currently identified m6A RNA alteration in autoimmune diseases.

Autoimmune Disease	Cells/Tissues Studied	Analyzed Elements/Mechanisms	Observations	Ref.
SLE	PBMC	m6A enzymes Writers: METTL3, METTL14, WTAP Erasers: FTO and ALKBH5 Readers: YTHDF2	Decreased expression of METTL14, ALKBH5 and YTHDF2 associated with clinical features of SLE patients	[134]
	PBMC	m6A enzymes Writers: METTL3, METTL14, WTAP Erasers: FTO and ALKBH5 Readers: YTHDF2, Comparison between SLE, RA, HBV, TB patients	Decreased expression of ALKHB5 related to clinical symptoms; METTL3, WTAP and FTO as potential collaborators of autoreactivity in SLE	[135]
RA	PBMC, m6AVar database	GWAS m6A-associated SNPs	Detection of m6A-SNPs within genes in immune cells and m6A regulators encoded genes	[156]
	PBMC, LPS-stimulated THP-1	m6A writer: METTL3	LPS stimulation enhances expression and biological activity of METTL3, overexpression of METTL3 attenuates inflammation,	[157]
	PBMC	m6A enzymes Writers: METTL3, METTL14, WTAP Erasers: FTO and ALKBH5 Readers: YTHDF2	global m6A content increase and ALKBH5, FTO, YTHDF2 decrease in RA	[158]
MS	GEO database	Association between m6A-SNPs and gene expression	Identification of 13 m6A-SNPs, rs923829 in METTL21B and rs2288481 in DKKL1 gene and association with MS	[202]
Ps	Skin samples	m6A methylation pattern, DMR	Hypomethylated transcripts in psoriasis affected skin linked with Wnt signaling pathway	[160]

SLE—systemic lupus erythematosus, RA—rheumatoid arthritis, MS—multiple sclerosis, Ps—psoriasis, HBV—hepatitis B virus, TB—tuberculosis, GEO database—Gene Expression Omnibus database, DMR—differentially methylated RNAs, SNP—single nucleotide polymorphism.

**Table 3:** Selected epigenetic drugs of reviewed autoimmune diseases in animal models and experiments.

Drug	Mechanism of Action	Disease/Model of Study	Effect	Ref.
Azacytidine (5-azaC, AZA)	DNMT inhibitor	SLE/human isolated T cells (CD4+, CD8+)	Amelioration of SLE symptoms	[207]
		MS/murine EAE model	Suppression of CNS inflammation	[226]
		RA/murine model of proteoglycan-induced arthritis	Amelioration of autoimmune arthritis	[206]
Decitabine (5-aza-dC, DAC)	DNMT inhibitor	MS/murine EAE model	Improvement of disease course	[209,227]
		RA/murine model of type II collagen induced arthritis	Amelioration of the clinical condition, diminished production of Th1 and Th17 proinflammatory cytokines	[208]
Trichostatin A (TSA)	Histone deacetylase inhibitor (Pan HDAC inhibitor)	MS/murine EAE model	Reduction of spinal cord inflammation, demyelination, neuronal and axonal loss and amelioration of disability	[228]
		MS/murine EAE model	Amelioration of neurodegeneration, reduced number of neutrophils	[212]
		MS/murine EAE model	Reduction of migration of T cells to the spinal cord and improved clinical outcome	[213]
		RA/human RASFs	Proinflammatory cytokine suppression and induction of apoptosis	[210]
		RA/human hypoxic RAFLS	Reduction of cell viability and increased apoptosis	[211]
		Psoriasis/human CD4+ T cells	Prevention of Treg differentiation into Th17 cells	[214]
Vorinostat (SAHA)	Histone deacetylase inhibitor (Pan HDAC inhibitor)	MS/human moDCs, murine EAE model	Inhibition of moDCs function (activation, maturation, antigen presentation); amelioration of CNS inflammation and demyelination	[229]
CKD-506	Selective histone deacetylase inhibitor	SLE/murine model of SLE	Decrease in the production of proinflammatory cytokines and improved renal outcomes	[230]
ACY-738	Selective histone deacetylase inhibitor	SLE/murine model of SLE	Decrease in B cell activation signaling pathways and reduction of PC differentiation	[231]
miRNA sponges	miRNA depletion	MS/cell culture/murine EAE model	Reduced number of Th17 cells	[232]

EAE—experimental autoimmune encephalitis, RASFs—rheumatoid arthritis synovial fibroblasts, RAFLS—rheumatoid arthritis fibroblast-like synoviocytes, moDCs—monocyte-derived dendritic cells, PC—plasma cells.

## Data Availability

No new data were created or analyzed in this study. Data sharing is not applicable to this article.
